# "QE = LIFE": The 20-Watt Paradox and the Gödelian Limitation of Biological Quantum Coherence

**DOI:** 10.7759/cureus.115578

**Published:** 2026-09-01

**Authors:** Atsuo Mori

**Affiliations:** 1 Cardiovascular Surgery, Kawasaki Municipal Hospital, Kawasaki, JPN

**Keywords:** entanglement, gödelian limitation, quantum, quantum coherence effects, quantum decoherence, quantum electrodynamics, yang-mills field equation

## Abstract

The human brain operates at an estimated power consumption of merely 20 Watts while performing massively parallel biological computations, a thermodynamic efficiency that classical silicon-based computers and standard non-relativistic quantum mechanics (for example, Schrödinger-based localized molecular states subject to rapid thermal decoherence) fail to explain.

This paper presents a foundational hypothesis: living systems sustain macro-scale quantum coherence and quantum entanglement (QE) in wet physiological environments (e.g., ~37°C aqueous cellular conditions) through non-Abelian Yang-Mills gauge fields and quantum electrodynamics (QED) phase dynamics. Traceable through a 60-year historical trajectory from Einstein’s skepticism to Clauser, Aspect, and Zeilinger’s experimental proofs (violating Bell's Inequality), QE demonstrates non-local behaviors where entangled entities maintain or even intensify their correlation regardless of spatial separation. Furthermore, while Abelian *U(1)* QED interactions are bounded by photon exchange at the speed of light in a vacuum (c), non-Abelian Yang-Mills gauge geometry enables instantaneous, simultaneous phase coordination across macroscopic biological networks (zero time-delay topological phase synchronization, *Δ t = 0*). We address the biological "Gödelian Limitation" - the epistemological impossibility of directly observing biological quantum coherence without inducing decoherence and death - and propose the existence of mathematically undecidable "Quantum Decoherence Diseases."

## Editorial

Introduction and the 20-watt paradox

Modern classical computing systems require megawatts of power to emulate complex neural network architectures. In contrast, the human central nervous system performs high-dimensional cognitive processing, motor control, and physiological orchestration under an energetic budget of approximately $P \approx 20\,\mathrm{W}$ [1]. Classical neurobiology attempts to explain this hyper-efficiency via chemical neurotransmission and ionic membrane action potentials governed by Na⁺/K⁺-ATPase pump dynamics [2]. However, signal propagation delays across synaptic clefts (1-5 ms) and chemical diffusion rates cannot account for the instantaneous, systemic synchronization observed across distant cortical structures.

Conventional quantum biology governed by the Schrödinger equation asserts that biological systems undergo near-instantaneous thermal decoherence within 10⁻¹³ seconds at physiological temperature ($T \approx 310\,\mathrm{K}$). We argue that this paradox arises from an overly reductive physical model. By incorporating quantum electrodynamics (QED) and Yang-Mills field theories, we demonstrate how living systems achieve phase synchronization with zero signal delay across macroscopic distances.

The 60-year historical arc of quantum entanglement (QE) (1935-1997)

To establish QE as the fundamental physical engine of biological architecture - a working paradigm we designate as "QE = LIFE" - we must trace the 60-year historical trajectory through which QE evolved from a rejected theoretical anomaly into an empirically validated law of nature.

In 1935, Einstein, Podolsky, and Rosen introduced the EPR paradox, dismissing entanglement as "spooky action at a distance" (*spukhafte* *Fernwirkung*) and insisting quantum mechanics was incomplete without local hidden variables [3]. In the same year, Schrödinger coined the term Verschränkung (entanglement) in response to EPR, recognizing it not as a minor feature, but as the characteristic trait of quantum mechanics that enforces non-separability [4]. Decades later, in 1964, Bell formulated Bell’s Theorem and Bell’s Inequality [5]. Under local realism, the expectation correlation parameter S is mathematically bounded by: *|S| = |E(a,b) - E(a,b') + E(a',b) + E(a',b')| ≤ 2* where a, a' and b, b' represent the different detector orientation settings (e.g., polarization angles) chosen by independent measurement stations, and E(a,b) denotes the statistical correlation coefficient between paired measurements at settings a and b. Quantum mechanics predicts that *S* can reach $2\sqrt{2} \approx 2.828$. A violation where *|S| > 2* provides a testable mathematical criterion to disprove local hidden variables, serving as definitive empirical proof of non-local QE.

In 1972, Freedman and Clauser conducted the world's first experimental test of Bell's Inequality using polarization-entangled photon pairs from atomic calcium cascades, observing measured correlation values that exceeded the classical upper mathematical limit * |S| ≤ 2 *, reaching * |S| > 2-a * (violating the CHSH inequality bound and approaching the quantum theoretical limit of 2*sqrt(2) approx 2.828) - a historical milestone known as the first empirical "violation" of Bell's Inequality - thereby providing the first direct experimental refutation of classical local hidden-variable theories [6]. Subsequently, in 1982, Aspect et al. performed time-varying optical switching experiments with entangled photon pairs, effectively closing locality loopholes and firmly proving the non-local reality of quantum states [7]. Finally, in 1997, Zeilinger et al. realized experimental quantum teleportation, demonstrating the practical transmission and manipulation of entangled quantum states over macroscopic distances - a milestone culminating in the 2022 Nobel Prize in Physics awarded to Clauser, Aspect, and Zeilinger [8].

Physical foundation: non-invasive distance coupling, QED, and Yang-Mills gauge dynamics

Unlike classical inverse-square physical forces, such as gravity or electrostatic Coulomb interactions that decay rapidly as distance increases $1/r^2$, QE exhibits a counter-intuitive non-local property: spatial separation does not weaken the non-classical correlation between entangled quanta. In an intriguing physical sense - analogous to a macroscopic "long-distance relationship" - the quantum non-separability remains fully intact or even appears relatively dominant over ambient local noise as physical separation increases.

To mathematically govern these interactions within biological tissue, we integrate QED and non-Abelian Yang-Mills gauge field theory. Under QED field theory, which is governed by Feynman path integrals and *U(1)* gauge symmetry [9], matter fields interact with the quantized vacuum electromagnetic field. This interaction induces macroscopic coherence domains (CDs) within biological water (H₂O) spanning micrometers to millimeters. However, standard QED interactions are fundamental electromagnetic processes mediated by gauge bosons (photons), which are strictly bounded by the finite propagation velocity of light in vacuum ($c \approx 3 \times 10^8$) - thereby introducing spatial latency.

To achieve systemic, zero-delay (*Δ t = 0*) coherence across the entire organism, biological architecture utilizes higher-order non-Abelian gauge symmetries (*SU(N)*) described by the Yang-Mills field equations [10]: $D_\mu F^{\mu\nu} = J^\nu, \quad \text{where } D_\mu = \partial_\mu - ig[A_\mu, \cdot]$ and the non-Abelian field strength tensor $F^{\mu\nu}$ is explicitly defined as: $F^{\mu\nu} = \partial_\mu A_\nu - \partial_\nu A_\mu - ig[A_\mu, A_\nu]$.

In these formulations, $D_\mu$ represents the gauge-covariant derivative, $A^\mu = A^\mu_a T^a$ denotes the non-Abelian gauge potential vector ($T^a$ being the generators of the Lie algebra *SU(N)* satisfying $[T^a, T^b] = i f^{abc} T^c$), *g* is the gauge coupling constant, $J^\nu$ is the conserved matter current density, and $[A^\mu, A^\nu]$ represents the non-zero Lie algebra commutator.

Because the tensor $F^{\mu\nu}$ includes these non-linear commutator terms ($[A^\mu, A^\nu] \neq 0$), the gauge fields interact directly with themselves. At the non-perturbative level, this cubic and quartic field self-coupling leads to a non-trivial vacuum topology characterized by non-zero topological invariants (e.g., Chern-Simons forms and Instantons/BPST configurations). This non-Abelian topology generates an instanton-mediated, topologically protected non-local manifold. Consequently, organismic coordination operates not via conventional wave-propagating photon exchanges (*Δ t = r/c*), but through a global, topologically condensed vacuum state that supports instantaneous, phase-locked non-local correlations (*Δ t = 0*) across the multi-layered biological architecture.

Unlike the Abelian *U(1)* symmetry of standard QED, where the field components commute ($[A^\mu, A^\nu] = 0$) and signal propagation is strictly bounded by the luminal speed limit *c*, the non-vanishing commutator $[A^\mu, A^\nu] \neq 0$ endows the non-Abelian gauge field with non-linear self-interactions.

At the non-perturbative level, this cubic and quartic field self-coupling leads to a non-trivial vacuum topology characterized by non-zero topological invariants (e.g., Chern-Simons forms and Instantons/BPST configurations). The gauge-invariant vacuum state establishes an instanton-mediated, topologically protected non-local manifold. Consequently, the organismic coordination operates not via conventional wave-propagating photon exchanges (*Δ t = r/c*), but through a global, topologically condensed vacuum state that supports instantaneous, phase-locked non-local correlations (*Δ t = 0*) across the multi-layered biological architecture.

Crucially, highly ordered biological structures act as non-linear transducers that bridge quantum-level gauge fluctuations with macro-level physiological functions. Metabolic energy flow continuously drives these biological architectures into a coherent, topologically protected steady state, thereby bypassing rapid thermal decoherence. Through this multi-scale structural coupling, distant biological macromolecules, organelles, and cortical areas collaborate in real-time through this simultaneous Yang-Mills quantum channel.

Epistemological barrier: the Gödelian limitation of LIFE

A fundamental question arises: if macroscopic *QE = LIFE* powers living organisms, why has it not been directly quantified in vivo? We term this barrier the Gödelian Limitation of Biological Coherence. In mathematical logic, Gödel’s Incompleteness Theorem demonstrates that a consistent formal system cannot prove its own consistency from within [11]. In quantum biology, the act of "observation" requires injecting measurement energy (*Δ E*) into the non-Abelian gauge domain. This intervention disrupts the delicate topological coherence field, reducing the biological system to a classical, thermal equilibrium state that no longer supports dynamic life processes. Thus, biological quantum coherence possesses an intrinsic observational limit in a living state.

Pathophysiological hypothesis: quantum decoherence diseases and Turing undecidability

In clinical medicine, numerous pathologies exhibit severe functional disruption despite normal structural MRI, genomic sequencing, and histopathology. We hypothesize that a distinct category of human pathology originates not from anatomical or molecular lesions, but from the localized collapse of Yang-Mills quantum coherence, termed "Quantum Decoherence Disease." Furthermore, determining whether a specific patient's disease stems from unobservable quantum decoherence or an undiscovered classical molecular defect is undecidable. If the gauge-invariant topological vacuum serves as the ultimate coordinator of biological homeostasis, then the degradation of this non-local coherence field must manifest as specific pathophysiological states-termed here as "Quantum Decoherence Diseases." 

Rather than conventional structural lesions, this concept may apply to idiopathic and sudden functional collapse, such as idiopathic ventricular fibrillation or unexplained lethal arrhythmias, where macro-scale phase-locking collapses instantaneously without prior anatomical breakdown. Similarly, functional network desynchronization in complex biological systems may represent a biological transition toward a state of "Turing Undecidability," where local algorithmic cellular processes become disconnected from global topological boundary conditions. By applying Turing’s Halting Problem [12] and Church’s Decision Problem (*Entscheidungsproblem*) [13], determining whether a specific patient's disease stems from unobservable quantum decoherence or an undiscovered classical molecular defect is undecidable.

Conclusion

The paradigm *QE = LIFE* unifies the thermodynamic miracle of the 20-watt human brain, non-Abelian gauge physics, and clinical medicine. Accepting the Gödelian and Turing limits of biological observation allows medicine to move beyond naive reductionism, recognizing that life's core essence resides in the unobservable, topologically protected quantum domain.
